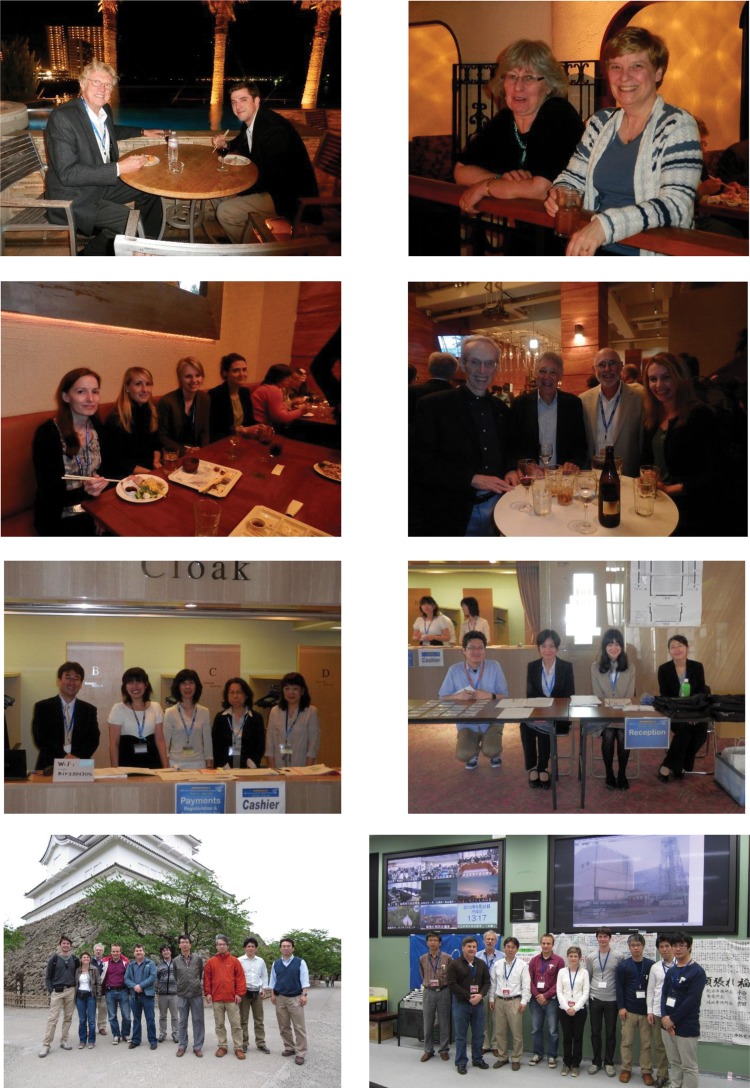# Preface

**DOI:** 10.1093/jrr/rru004

**Published:** 2014-03

It was our great honor and pleasure that we hosted the Heavy Ion Therapy and Space Radiation Symposium 2013 (HITSRS2013) on May 15^th^ to 18^th^, 2013 in Chiba, Japan. The symposium focused on research in heavy ion radiotherapy and the effects of space radiation in low earth orbit. Many researchers in the field of biology, medicine and physics participated in the symposium and made oral and poster presentations. More than 220 participants including 130 from abroad participated in this symposium.

Heavy ion cancer therapy is a highly effective form of radiation treatment for a variety of malignant tumors. Successful clinical results from heavy ion treatment facilities throughout world have led to a growing interest in this modality and there was much active discussion during the sessions devoted to heavy ion cancer therapy. The Heavy Ion Medical Accelerator in Chiba (HIMAC) at the National Institute of Radiological Sciences (NIRS) is one of the leading heavy ion cancer therapy facilities and has treated over 7000 patients to date. There were many presentations of the biological research carried out at heavy ion accelerator facilities throughout the world including HIMAC in Japan, NSRL in USA, Heidelberg and GSI in Germany, Lanzhou in China and elsewhere. Space radiation research is also a big challenge because the radiation environment in space is very complex. The space radiation environment is unique with a significant component of high Z, high energy ions as well as other types of radiation including energetic protons, neutrons, and gamma-rays. There were many presentations of biology and physics research using the above heavy ion accelerators to simulate the space radiation environment. In addition, several presentations reported their results from space experiments carried out on the International Space Station (ISS).

We consider the timely publication of the proceedings of the symposium to be important to share the information with scientists and experts all over the world. The proceedings will provide valuable information for better understanding of heavy ion radiotherapy and space radiation.

December 2013


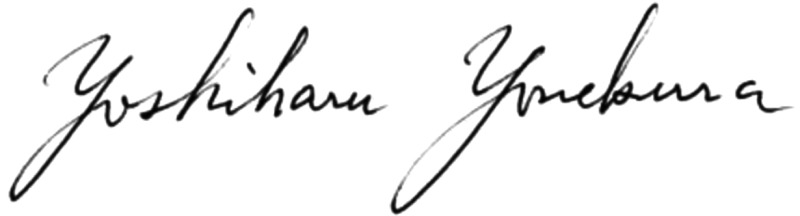


Yoshiharu Yonekura MD, Ph.D.

President of HITSRS2013

**Figure:**
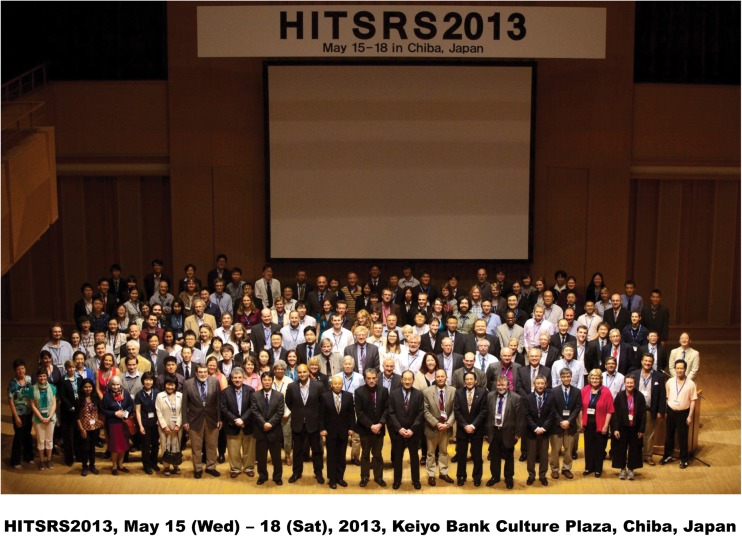

**Figure:**
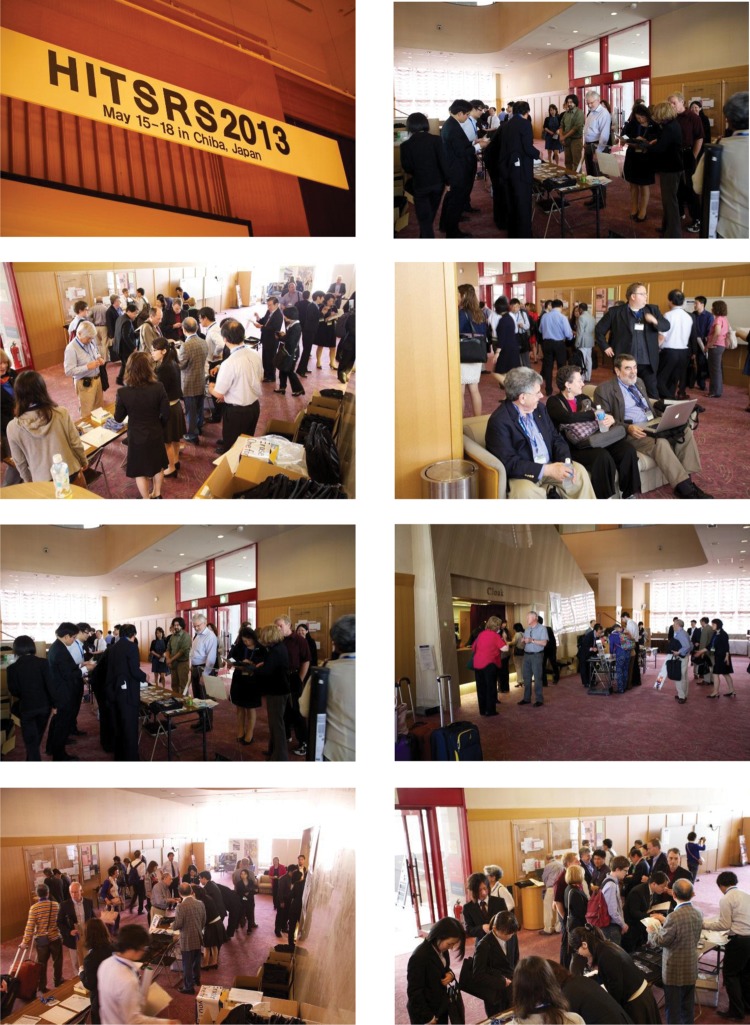

**Figure:**
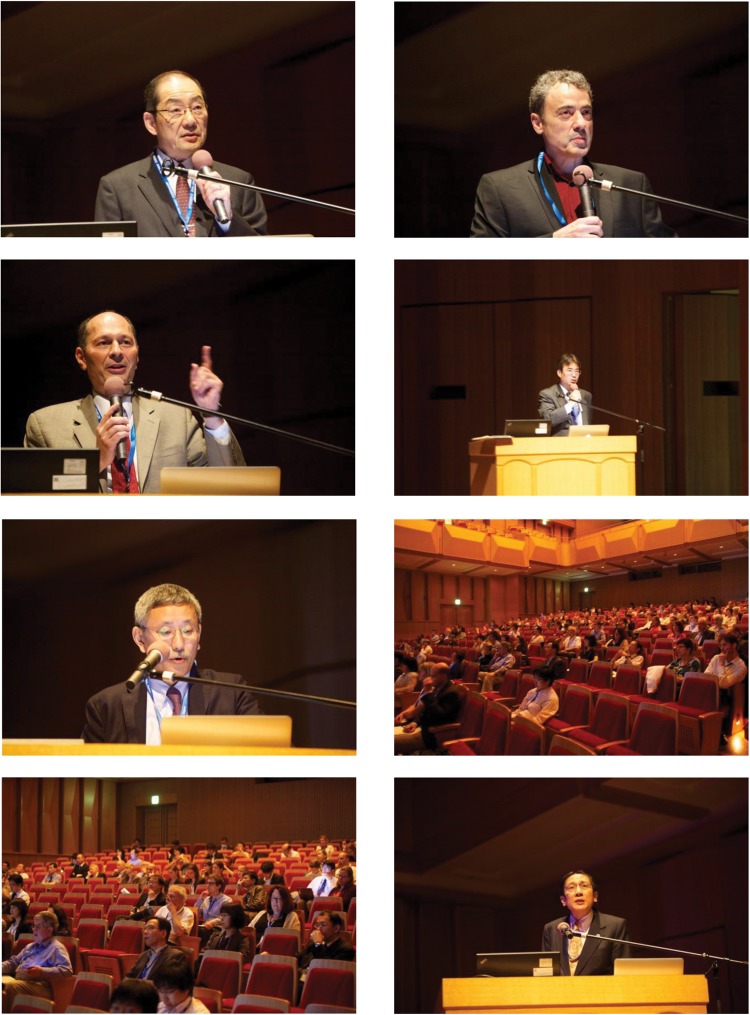

**Figure:**
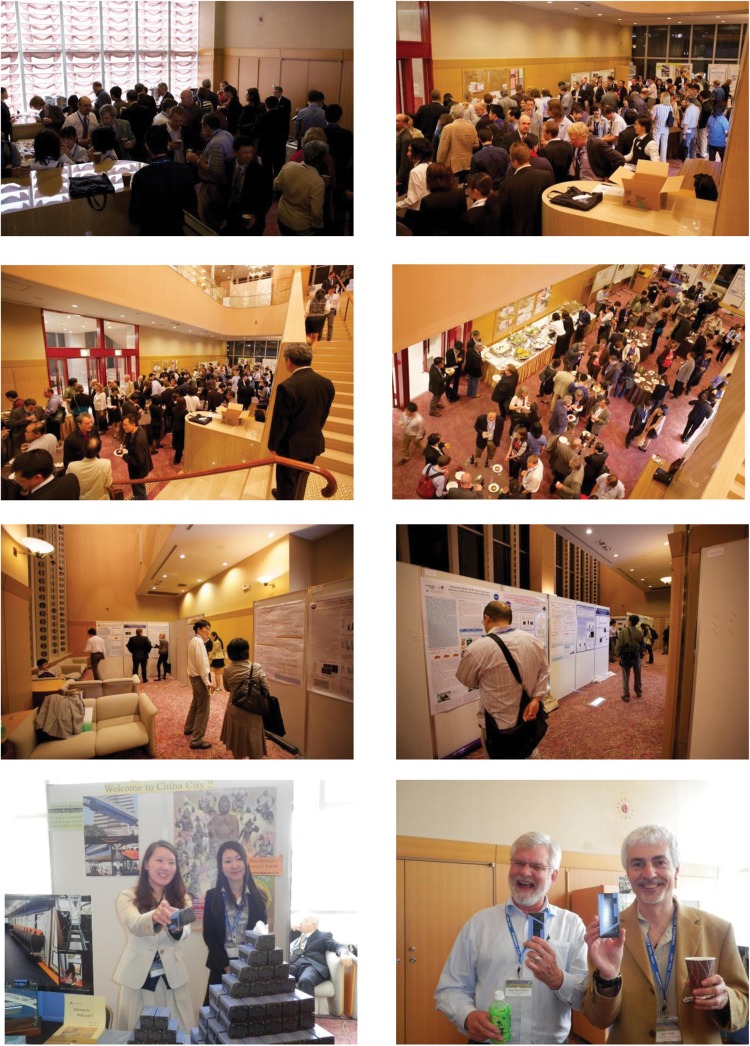

**Figure:**
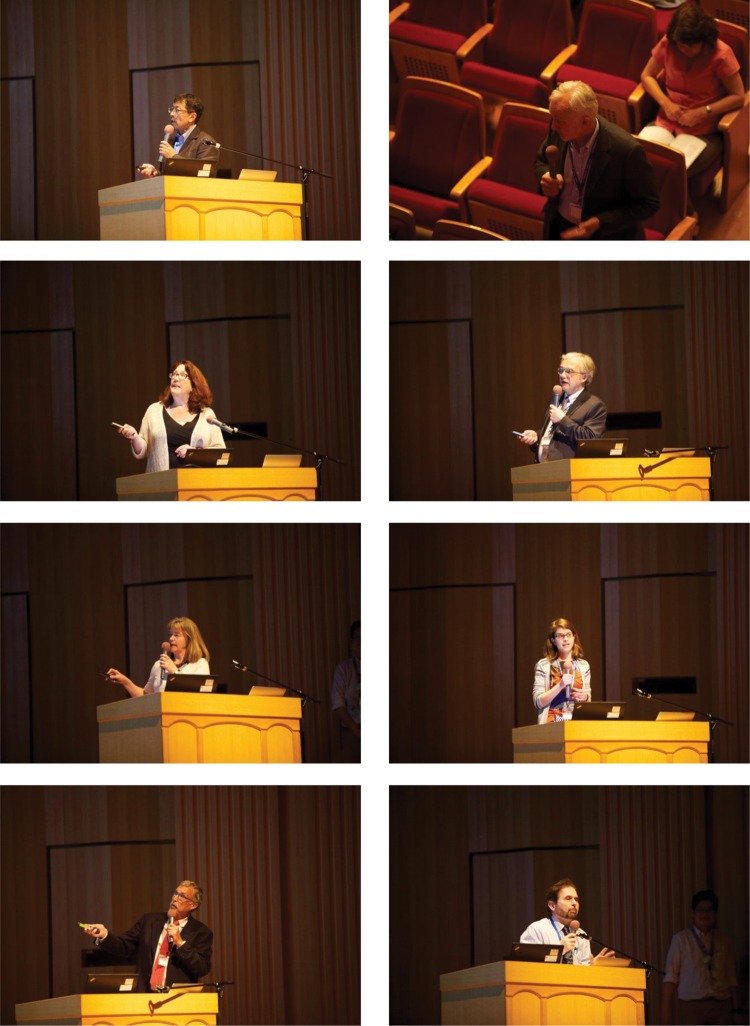

**Figure:**
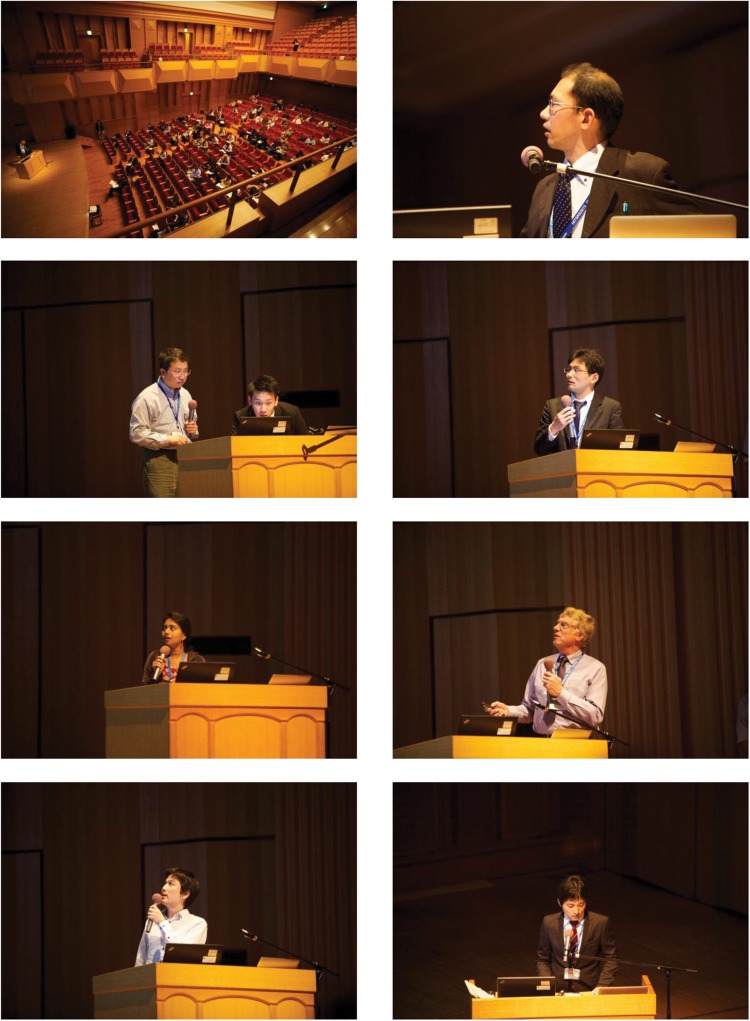

**Figure:**